# Parallel Process Latent Growth Modeling of the Relationships Between Social Cohesion and Mental Health in Later Life

**DOI:** 10.1093/geroni/igaa057.983

**Published:** 2020-12-16

**Authors:** Sok An, Kyeongmo Kim, Minhong Lee

**Affiliations:** 1 Korea Rural Economic Institute, Austin, Texas, United States; 2 Virginia Commonwealth University, Richmond, Virginia, United States; 3 Dong-eui University, Busan, Pusan-jikhalsi, Republic of Korea

## Abstract

Previous literature suggests that social factors (e.g., social cohesion, social support) are protective predictors of mental health problems. However, there might be a reciprocal relationship between social factors and mental health and the relationship changes over time. Therefore, this study examined the longitudinal relationship between community social cohesion and mental health using a latent growth curve model with 8 waves of the National Health and Aging Trends Study (NHATS; 2011-2018), a nationally representative panel study of Medicare beneficiaries in the United States. Social cohesion measured the perceived level of mutual trust by three items (score range: 0-6) and mental health was measured by PHQ-4 (score range: 0-12). The final model including covariates (age, gender, functional disabilities) fit the data well: χ2=1036.383, p<.001; RMSEA=.037; CFI=.960; and SRMR=.070. Initial level of social cohesion was negatively associated with initial level of mental health problem (β=−.23, p< .001), suggesting that higher levels of social cohesion was associated with lower levels of mental health problems. The covariance between social cohesion slope and mental health slope was significant (β=−.16, p< .01), suggesting an increase in social cohesion was associated with a decrease in mental health problems over time. Functional disabilities significantly influenced mental health over time, while functional disabilities did not influence social cohesion consistently. This study adds to the growing literature on the ways mental health status and social connection have reciprocal relationships over time. Therefore, mental health status in later life could be decreased by improving social cohesion and connectedness with the community.

